# Supplementary material for: A split ALFA tag-nanobody system for protein localization and proximity proteomics in mycobacteria
Source: mBio. 2025 Jun 27;16(8):e00971-25. doi: 10.1128/mbio.00971-25 (PMC12345155; doi:10.1128/mbio.00971-25)
Supplement: Supplemental material — Figures S1-S4 and supplemental table captions. [file mbio.00971-25-s0001.pdf]

Supplementary Materials

Figures S1-S4

Table S1: Strains, plasmids, oligos, and DNA sequences used in this study.

Table S2: RpoC-ALFA vs no ALFA control proximity data. Analysis of biotinylated peptides from proximity labeled lysates made from *M. smegmatis* strains MGM7106 and MGM7085.

Table S3: PKS13-ALFA (4 tag positions) vs no ALFA control proximity data. Analysis of biotinylated peptides from proximity labeled lysates made from *M. tuberculosis* strains MGM7416, MGM7418, MGM7419, MGM7420, and MGM7421.

Table S4: 15 sample proteins involved with mycolic acid synthesis and transport. Log_2FC and Adjusted P-values for all 4 ALFA tag positions shown. Highlighted in green are $Log_2FC>1$.

Table S5: List of PKS13-ALFA proximity hits with $Log_2FC>1$ and adjusted P-value<0.0001 used for generating Venn diagram (Figure 6D).

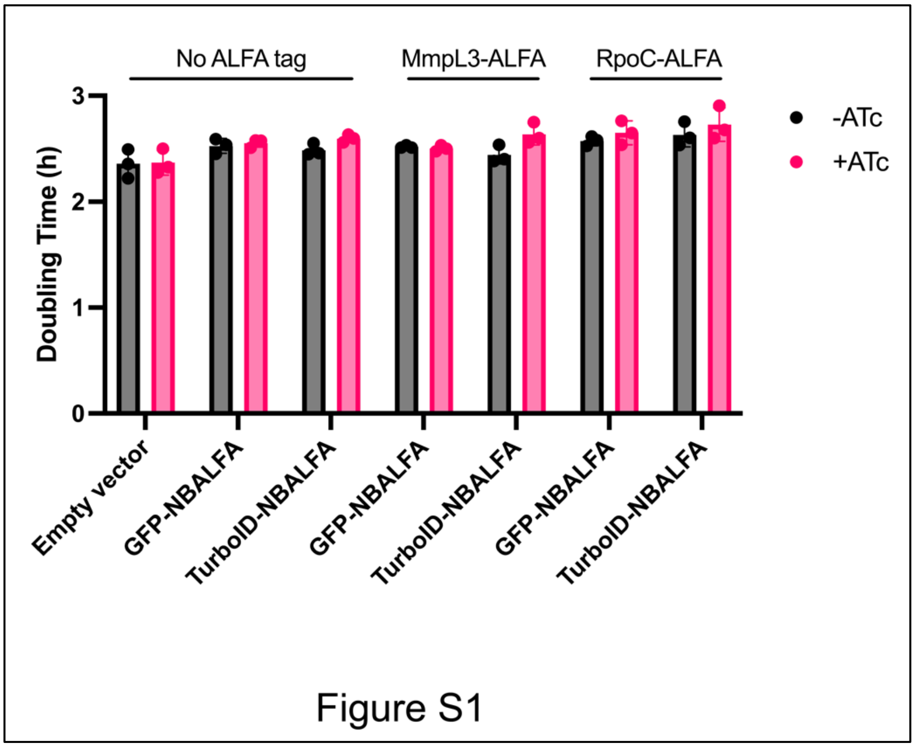

Figure S1. Doubling times of inducible NBALFA expression strains with and without ALFA tagged proteins. Strains were grown in the absence and presence of inducer (ATc, 50ng/ml). Each graphed point is an independently calculated doubling time from a growth curve representing three technical replicates per time point. Error bars are SD.

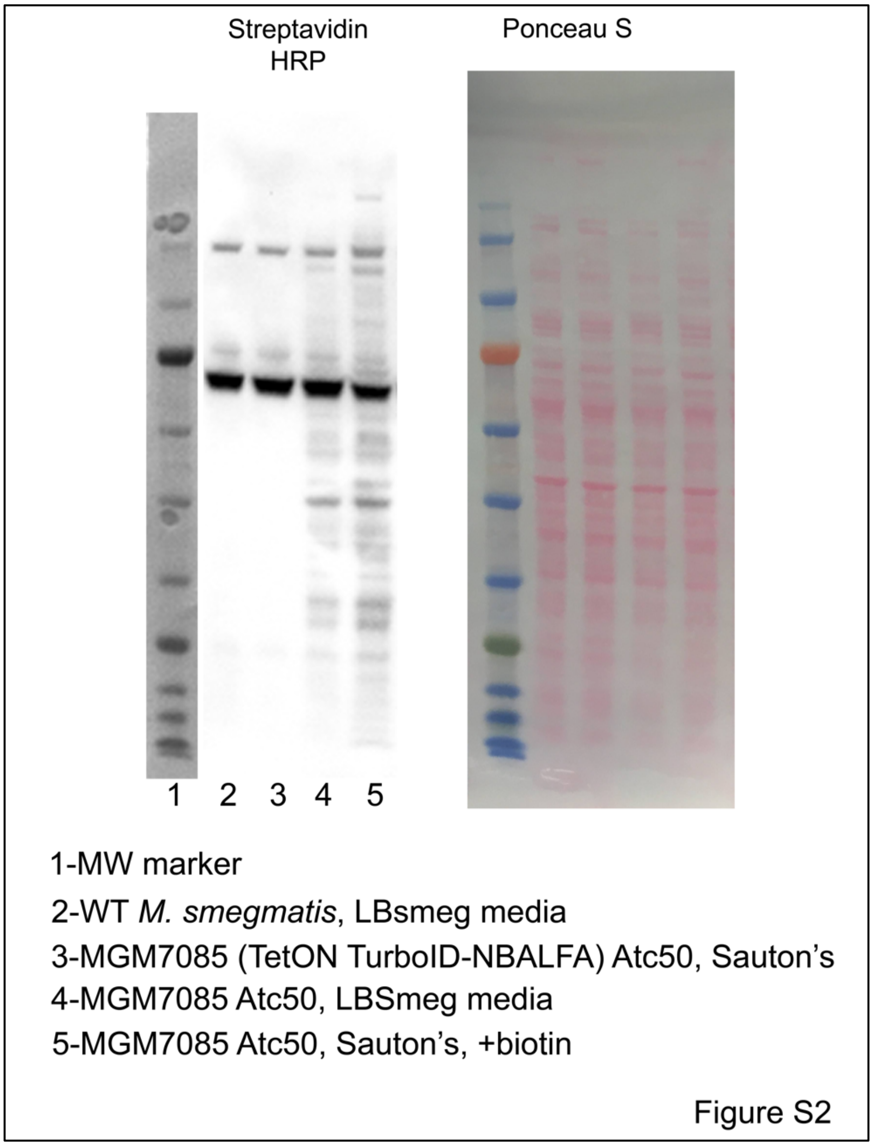

Figure S2

Streptavidin HRP blot (left) and ponceau S staining (right) of the indicated strains and growth conditions showing levels of endogenous biotinylated proteins in *M. smegmatis*.

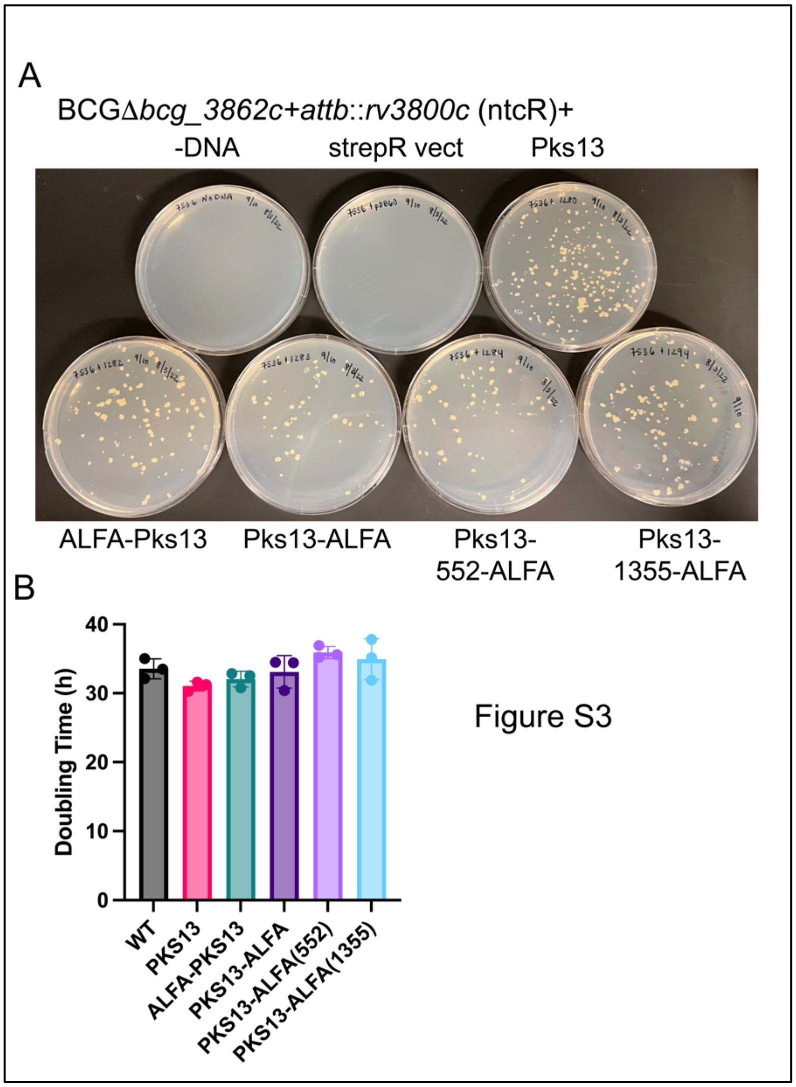

Figure S3

Functionality testing of PKS13-ALFA alleles via *attB* allelic exchange and doubling times of resulting strains.

(A). Shown are streptomycin agar media with transformation of a merodiploid BCG strain with a chromosomal deletion of *pks13* (*BCG_3862c*) and a second copy of *M. tuberculosis rv3800c* at the *attB* site conferring nourseothricin-resistance. Transformation of this strain with an *attB* integrating vector conferring streptomycin-resistance encoding nothing (strepR vect) or alleles of *pks13* with no ALFA tag, or four different ALFA tag position in the PKS13 protein.

(B) Doubling times calculated for strains in (A) grown in liquid culture with wildtype BCG as control. Each graphed point is an independently calculated doubling time from a growth curve representing one independent culture. Error bars are SD.

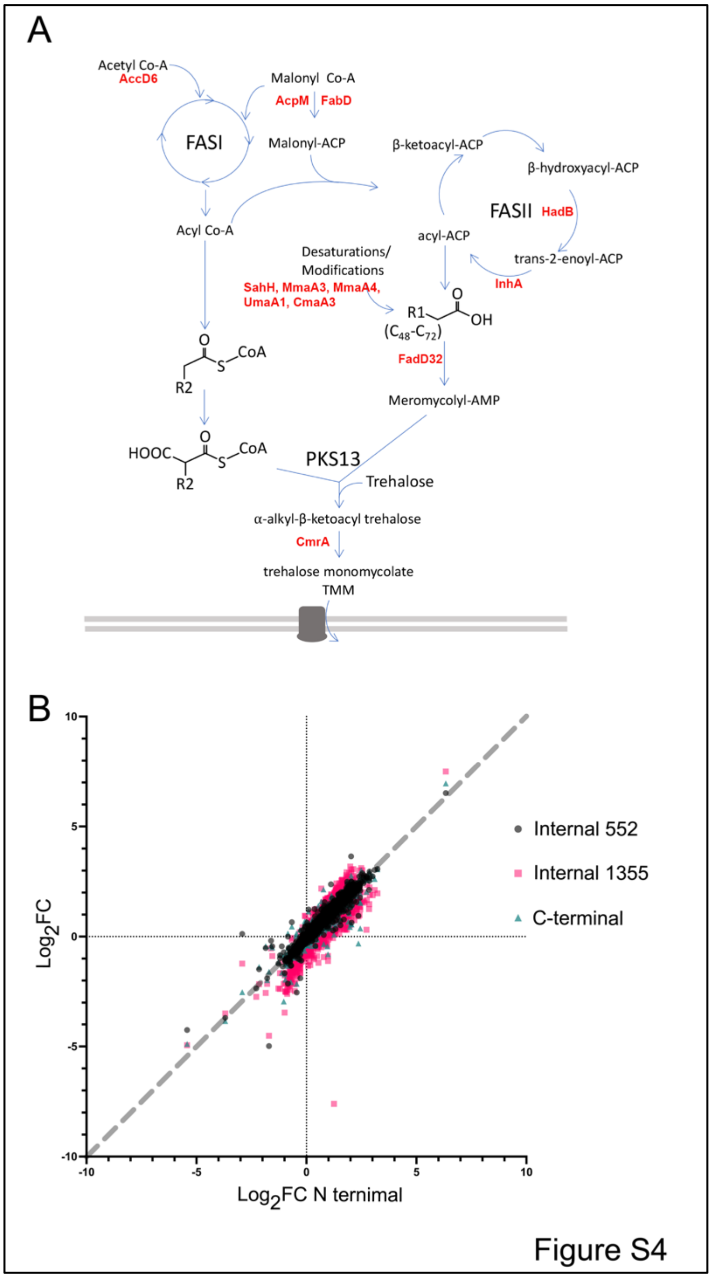

Figure S4. PKS13-ALFA proximity labeled hits within the mycolic acid biosynthesis pathway. (A) Proteins found in all four ALFA tagged labeling conditions with a Log_2FC >1.3 and adjusted P-value < 0.05 in bold, red fonts superimposed on the mycolate biosynthetic pathway **(B)** Scatter plot of Log_2FC of PKS13-ALFA tagged proximity labeling vs No ALFA controls by tag position. N-terminal ALFA is plotted on X-axis and compared to C-terminal, Internal 552, or Internal-1355 ALFA tags plotted on Y-axis.
